# Acetylcholine Muscarinic Receptors in Ventral Hippocampus Modulate Stress-Induced Anxiety-Like Behaviors in Mice

**DOI:** 10.3389/fnmol.2020.598811

**Published:** 2020-12-15

**Authors:** Li Mei, Yu Zhou, Yi Sun, Hong Liu, Dengwen Zhang, Pingping Liu, Haihua Shu

**Affiliations:** ^1^Department of Anesthesiology, Guangdong Provincial People’s Hospital, Guangdong Academy of Medical Sciences, Guangzhou, China; ^2^Department of General Surgery, Guangdong Provincial People’s Hospital, Guangdong Academy of Medical Sciences, Guangzhou, China; ^3^The Second School of Clinical Medicine, Southern Medical University, Guangzhou, China

**Keywords:** chronic stress, anxiety, hippocampus, acetylcholine muscarinic receptors, scopolamine, electrophysiology

## Abstract

Chronic stress exposure increases the risk of developing various neuropsychiatric illnesses. The ventral hippocampus (vHPC) is central to affective and cognitive processing and displays a high density of acetylcholine (ACh) muscarinic receptors (mAChRs). However, the precise role of vHPC mAChRs in anxiety remains to be fully investigated. In this study, we found that chronic restraint stress (CRS) induced social avoidance and anxiety-like behaviors in mice and increased mAChR expression in the vHPC. CRS increased the vHPC ACh release in behaving mice. Moreover, CRS altered the synaptic activities and enhanced neuronal activity of the vHPC neurons. Using pharmacological and viral approaches, we showed that infusing the antagonist of mAChRs or decreasing their expression in the vHPC attenuated the anxiety-like behavior and rescued the social avoidance behaviors in mice probably due to suppression of vHPC neuronal activity and its excitatory synaptic transmission. Our results suggest that the changes of neuronal activity and synaptic transmission in the vHPC mediated by mAChRs may play an important role in stress-induced anxiety-like behavior, providing new insights into the pathological mechanism and potential pharmacological target for anxiety disorders.

## Introduction

Maladaptive responses to chronic stress are associated with the onset and exacerbation of emotional abnormalities, including anxiety disorders and social avoidance (Dias-Ferreira et al., 2009; McEwen and Morrison, 2013; McEwen et al., 2015; Sandi and Haller, 2015). It is reported that more than 30% of adults and adolescents present an anxiety disorder in their lifetime and that 25%–80% of patients admitted to the hospital for surgery experienced preoperative anxiety (Bluett et al., 2014; Kassel et al., 2018). The anxiety impacts negatively influence the quality of life of patients. Furthermore, during postsurgical recovery, anxiety negatively influences the intensity of postoperative pain and analgesia requirement and even increases postoperative morbidity and mortality in certain types of surgery (Kassel et al., 2018). Various treatments, such as acceptance and commitment therapy, cognitive therapy, music therapy, and pharmacological interventions, have been applied to patients suffering from anxiety disorder. However, currently available treatments for anxiety disorders are inadequate. For instance, the drugs lorazepam and pregabalin, intended to treat anxiety, have failed to decrease preoperative anxiety and postoperative pain (Kassel et al., 2018). Currently, mechanisms underlying anxiety need to be fully understood for developing adequate treatments.

Previous studies in rodents have shown that the ventral hippocampus (vHPC) is involved in regulating anxiety-like behavior. Bilateral lesions of the vHPC reduce mouse anxiety-like behavior (Bannerman et al., 1999, 2003; Weeden et al., 2015), whereas other studies using optogenetic or pharmacological stimulation of the region found the opposite effect (Felix-Ortiz et al., 2013; Padilla-Coreano et al., 2016). Using freely moving calcium imaging, a recent study in mice reported anxiety cells in ventral CA1 (vCA1) region (Jimenez et al., 2018). Acute activation of vCA1 neurons induces anxiety-like behaviors in mice; however, little is known about molecular and synaptic mechanisms underlying the changes of these neuronal activities in this region after chronic stress.

In the central neuronal system (CNS), acetylcholine (ACh) facilitates many functions, such as learning, memory, attention, and motor control (Hasselmo, 2006; Thomsen et al., 2007; Wess et al., 2007). ACh muscarinic receptor (mAChR) family is one of the two ACh receptors, with subtypes M1, M2, M3, M4, and M5 (Volpicelli and Levey, 2004). They are expressed in many regions of the CNS including the prefrontal cortex, amygdala, and hippocampus. In the hippocampus, M1 is the predominant receptor (approximately 60%) while M2 and M4 are less abundant (approximately 20%). Throughout the hippocampus, M1 is expressed mainly in the pyramidal cell bodies and apical and basal dendrites of the stratum radiatum and stratum oriens. It has been recently demonstrated that mAChRs play a role in learning and memory (Bartko et al., 2014; Dasgupta et al., 2018; Felder et al., 2018). Unbalanced ACh levels in the hippocampus cause abnormal emotional behaviors (Mineur et al., 2013). Inescapable stress can elevate ACh levels in the hippocampus (Mark et al., 1996). Increased ACh levels by inhibiting acetylcholinesterase (AChE) in the hippocampus of mice induce anxiety-like and depression-like behaviors (Mineur et al., 2013). However, the functional plasticity of mAChRs in stress-induced mood disorders remains unresolved.

In this study, we investigated the role of vHPC mAChRs in stress-induced anxiety behaviors. Our results suggest that increased expression of mAChRs in the vHPC contributed to stress-induced anxiety-like behavior *via* promoting excitatory synaptic transmission in the vHPC.

## Materials and Methods

### Animals

This study was approved by the Institutional Laboratory Animal Care and Use Committee of Guangdong Provincial People’s Hospital. All of the experiments were implemented according to the rules of the Chinese Council on Experimental Animal Care. In all of the experiments, male C57BL/6J mice (8–9 weeks, four per cage) were housed with a 12-h light/dark cycle (lights on at 8:00 a.m. and lights off at 18:00 p.m.) under standard conditions (21°C–26°C). Behavioral tests were performed from 10:00 to 15:00, and animals’ behaviors were monitored with video tracking software (Ethovision XT10, Noldus). Animal suffering was minimized through our efforts.

### Chronic Restraint Stress

Male mice were restrained in well-ventilated Perspex tubes for 2 h (between 8:00 a.m. and 12:00 p.m., without water and food) for 10 consecutive days. Twenty-four hours after restraint stress, mice were subjected to behavioral tests, Q-PCR, Western blotting assessments, electrophysiological recording, and immunofluorescence staining.

### Intracerebral Infusions and Stereotaxic Injection

Animals were anesthetized using pentobarbital (intraperitoneal, 50 mg/kg) and mounted onto a mouse stereotaxic frame. A small hole in the skull was made (1 mm diameter) using a dental drill. A stainless steel guide cannula (Plastics One, Incorporation; C315G/SPC; length, 4.0 mm) was lowered into the vHPC according to the following coordinates: anterior, −3.0 mm; lateral, 3.6 mm; ventral, −3.4 mm from the Bregma. The guide cannula was fixed in place using glass ionomer cement. Mice were placed back into their home cages to recover. All of the behavioral experiment tests were performed 7 days after cannula placement. After removal of the dummy cannula, intra-vHPC injection was performed using an infusion cannula (Plastics One, Incorporation; C315I/SPC, length matched to the guide cannulas) connected to a 10-μl microsyringe (Hamilton, Reno, NV, USA) *via* polyethylene tubing (Plastics One, Incorporation; C313C). The injection volume was 0.5 μl/side at a rate of 0.1 μl/min, which was controlled by a microinfusion pump (RWD200, China). The infusion cannula was then held in place for 2 min to allow for diffusion of the drug before being replaced by the dummy cannulas.

We used shRNA, a gene silencing technology to genetically interfere with M1 mAChR expression. The M1shRNA has been previously described (Wohleb et al., 2016) and contains the sequence 5′-TGCAACGCCTCTGTCATGAATCTTCTTCAAGAGAGAAGATTCATGACAGAGGCGTTGCTTTTTTC-3′. The expression plasmid AAV-U6-M1shRNA-CMV-RFP was constructed by using molecular biological techniques and packaged into an adeno-associated virus (AAV9) at a titer of 1 × 10^13^ vg/ml. We used a microsyringe pump (Nanoliter 2000 Injector, WPI) to inject the virus (300 nl, 100 nl per min) into the vHPC according to the following coordinates: anterior, −3.0 mm; lateral, 3.6 mm; ventral, −3.7 mm from the Bregma.

### Fiber Photometry Recording

AAV-syn-GAch2.0 (a fluorescent ACh indicator, purchased from Vigene Bioscience, Shandong, China) virus was injected into the vHPC (titer, 1 × 10^12^ vg/ml, 300 nl), then, an optical fiber (200 μm outer diameter, 0.37 numerical aperture) was placed in a ceramic ferrule and inserted toward the vHPC through the craniotomy and fixed in place using glass ionomer cement. Mice were placed back into their home cages to recover for at least 2 weeks. Fluorescence signals were acquired with a fiber photometry system equipped with a 488-nm excitation laser, 505–544 nm emission filter and a photomultiplier tube (R3896, Hamamatsu). The laser power at the tip of the optical fiber was adjusted to the low level of ~30 mW to minimize bleaching.

For the fiber photometry experiments, mice were habituated to the testing house for 5 min. Then, mice were gently held by the experimenter for 10 s using the right hand to imitate transient restraint stress. Every mouse went through this stress six times with an interval of more than 60 s.

We calculated (*F* — *F*0)/*F*0 to obtain the fluorescence change value (Δ*F*/*F*), where *F*0 was the average value of baseline fluorescence signal in the 5-s control time window. Δ*F*/*F* values were represented by SEM or heat map with shaded areas, which represented signal changes over and over again.

### Social Interaction Test

The chamber consists of a large open field (89 × 63 × 60 cm) within a small wire cage (14 × 17 × 14.5 cm) at one side of it. Mice were habituated to the testing house at least 1 h before the test in low light lux condition (~50 lux). The test was divided into two phases. In the first phase, mice were running free in the chamber for 2.5 min. In the second phase, an unfamiliar male mouse was introduced into the wire cage. The length of time the mice spent in the interactive zone was recorded. We recorded the interaction time of the mice with the wire cage during two 2.5-min phases (with and without the unfamiliar C57BL/6J mouse). The ratio of the two phases is defined as the interaction index.

### Open-Field Test

Before the experiment, mice were habituated in the experiment room for 1 h, then mouse was placed in the center of the chamber (40 × 40 × 30 cm). A 25-watt halogen bulb is used to illuminate open field areas (~650 light lux). The mouse was gently placed and monitored by Versamax animal behavior monitoring (AccuScan, Columbus, OH, USA) for 5 min. Time mice spent in the center (15 × 15 cm square) and total distance were measured, and 75% ethanol was used to clean the chamber between trials.

### Elevated Plus Maze Test

The elevated plus maze (EPM) is located 50 cm above the ground. The maze consists of two open arms (25 × 8 × 0.5 cm) and two closed arms (25 × 8 × 12 cm) that extend from a central platform (8 × 8 cm). Closed arms are surrounded by transparent walls 12 cm high, and open arms are surrounded by 0.5-cm ledges with ~650 light lux centered over the open arms. Each mouse was placed in the center platform, facing a closed arm. During the 5-min test, cameras above the maze recorded the mice’s movements. The researchers measured the time mice spent in open or closed arms. The maze was cleaned with 75% ethanol between each test.

### Real-Time Quantitative PCR

After chronic restraint stress (CRS), mice went through the behavior tests to ensure the stress-induced anxiety. Total vHPC RNA was extracted from C57BL/6 mice using RNeasy Mini Kit (QIAGEN) according to the instructions. RNA concentrations were then determined *via* Nanodrop. RNA was reverse transcribed to cDNA using PrimeScript™ RT Reagent Kit (Takara RR037A, Japan). Real-time PCR was performed on a 7500 RT-PCR instrument using the SYBR^®^ Premix Ex Taq™ (RR420A, Takara, Japan). Relative expression levels were determined according to the ^ΔΔ^Ct method.

### Western Blot

Mice were anesthetized with isoflurane and transcardially perfused with phosphate buffered saline (PBS) buffer. Hippocampal samples were homogenized in radioimmunoprecipitation assay (RIPA) buffer (150 mM NaCl, 1% NP-40, 0.5% sodium deoxycholate, 0.1% sodium dodecyl sulfate (SDS), 1 mM ethylenediaminetetraacetic acid (EDTA), and 50 mM Tris HCl, pH 7.4) containing protease and phosphatase inhibitors and then incubated for 30 min on ice. The homogenates were centrifuged at 14,000× *g* for 30 min at 4°C, and then the supernatants were collected for Western blot. Protein concentrations were determined using bicinchoninic acid (BCA) assay (Thermo Fisher Scientific). RIPA extracted brain lysates (60–80 μg) were run on 10% SDS–polyacrylamide gel electrophoresis (PAGE) gel. Proteins were transferred onto polyvinylidene difluoride (PVDF) membrane and blocked in 5% nonfat dried milk in Tris-buffered saline with Tween 20 (TBST). The membrane was then incubated in the following antibodies: rabbit monoclonal anti-M1 (1:200; M9808; Sigma–Aldrich) and mouse monoclonal anti-glyceraldehyde 3-phosphate dehydrogenase (GAPDH; 1:10,000; MAP374; Millipore). Primary antibodies were detected with the species-specific horseradish peroxidase (HRP)-conjugated secondary antibody, and then visualized by enhanced chemiluminescence (ECL). Chemiluminescent signal was captured on an LAS4000 Fuji Imager. Densitometry analysis was performed using Quantity One software (Bio-Rad).

### Brain-Slice Electrophysiology

Slices were prepared as described previously (Tao et al., 2013). After being anesthetized with pentobarbital sodium (50 mg/kg), mice were decapitated and the brain was transferred into the ice-cold artificial cerebrospinal fluid (ACSF, cutting solution) made of 2.5 mM KCl, 220 mM sucrose, 2.5 mM MgSO_4_, 1.3 mM CaCl_2_, 1 mM NaH_2_PO_4_, 26 mM NaHCO_3_, and 10 mM glucose. Horizontal slices (300 μm) containing the vHPC were cut with a vibrating microtome (Leica VT 1000S, Germany). Slices were then moved into a chamber for 20 min recovery in 32°C and kept at room temperature for additional 1 h before recording. The chamber contained the recording ACSF, made of 26 mM NaHCO_3_, 126 mM NaCl, 1.2 mM NaH_2_PO_4_, 3 mM KCl, 2 mM CaCl_2_, 1 mM MgSO_4_, and 10 mM glucose. All solutions were saturated with 95% O_2_/5% CO_2_ (vol/vol).

Slices were transferred into a recording chamber, and the recording solution (ACSF) was perfused through the chamber at a flow rate of 3 ml/min. To record spontaneous excitatory postsynaptic currents (sEPSCs), pipettes filled with the intracellular solution composed of 30 mM KCl, 105 mM K-gluconate, 10 mM phosphocreatine, 10 mM 4-(2-hydroxyethyl)-1-piperazineethanesulfonic acid (HEPES), 4 mM ATP-Mg, 0.3 mM ethylene glycol-bis(β-aminoethyl ether)-N,N,N′,N′-tetraacetic acid (EGTA), 0.3 mM GTP-Na, and 5 mM QX314 (pH 7.4, 290 mOsm) were used. sEPSCs were recorded at a holding potential of −60 mV in the presence of bicuculline methiodide (BMI, 20 μM) for blocking GABAergic synaptic currents. Spontaneous inhibitory postsynaptic currents (sIPSCs) were recorded at a holding potential of 0 mV, with a pipette filled with an internal solution containing 140 mM CsCl, 2 mM MgCl_2_, 1 mM CaCl_2_, 10 mM EGTA, 10 mM HEPES-CsOH, 2 mM adenosine triphosphate, and 5 mM QX-314. Then, 1 mM kynurenic acid was added to the recording solution. Digidata1440 converter and Axoclamp-700B amplifiers (Molecular Devices, Sunnyvale, CA, USA) were used. For measuring firing frequency, steady-state current was injected in +20 pA increments from −60 to 220 pA. All action potential properties and excitability recordings were performed in the presence of 2,3-dihydroxy-6-nitro-7-sulfanilobenzene(f)quinoline-2,3-diketone (NBQX, 10 mM) and BMI (20 μM). All reagents were purchased from Sigma–Aldrich. All analyses were performed using Clampfit 10.2 (Axon Instruments/Molecular Devices), Minianalysis.

### Immunofluorescence

Mice were anesthetized using pentobarbital sodium (50 mg/kg), perfused with saline, followed by 4% methanol in 0.1 M phosphate buffer (PBS). After which brain tissue was removed, postfixed into 4% methanol for 6 h, and then immersed in 30% sucrose. After 3 days, a freezing microtome (Leica) was used to obtain 40-μm-thick coronal brain sections. Slices were then washed with PBS and treated with 1% Triton-100, followed by goat serum and incubation with c-fos primary antibody (Santa Cruz Biotechnology, -sc-52, 1:3,000) at 4°C overnight. Slices were then incubated with the corresponding fluorescence-conjugated secondary antibody [Alexa Fluor 488 (1:500, A11034, Invitrogen)] at room temperature for 2 h. Images with fluorescence were captured using a Nikon A1 confocal microscope with the resolution of 1,024 × 1,024 pixels and were then processed using National Institutes of Health (NIH) ImageJ software.

### Statistical Analyses

Statistical analysis was performed using SPSS software. An independent sample *t*-test or one-way analysis of variance (ANOVA) or two-way ANOVA was used for statistical analysis, followed by a least square difference (LSD) test for *post hoc* comparisons. All data are represented as mean ± standard error of the mean (SEM). The statistical significance level was set at *p < 0.05, **p < 0.01, ***p < 0.001.

## Results

### Chronic Restraint Stress Induces Social Avoidance and Anxiety-Like Behaviors and Increases Acetylcholine Muscarinic Receptor Expression in Ventral Hippocampus

**Figure 1:**
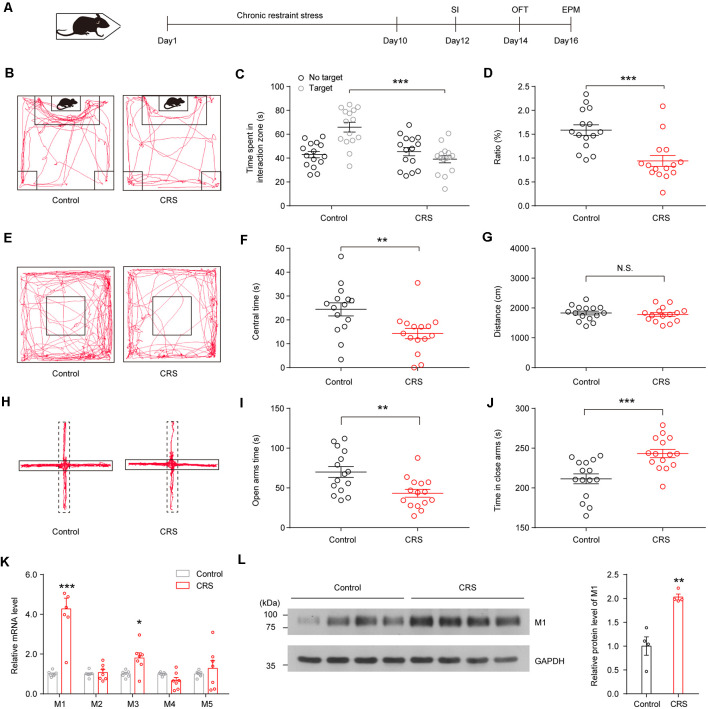
Chronic restraint stress (CRS) induces social avoidance and anxiety-like behaviors and increases acetylcholine (ACh) muscarinic receptor (mAChR) expression in ventral hippocampus (vHPC) in mice. **(A)** CRS procedure: mice were restrained for 2 h/day for 10 days and subsequently subjected to behavioral tests. **(B)** Representative tracks of control and stress group in social interaction (SI) test. **(C)** SI time in the absence or presence of social target. Two-way analysis of variance (ANOVA), *F*_(1,56)_ = 5.631, *p* < 0.001. **(D)** The ratio in the SI test was decreased in the CRS group. Unpaired two-tailed Student’s *t*-test, *t*_(28)_ = 4.086, *p* < 0.001. **(E)** Representative tracks of control and stress group in the open-field test (OFT). **(F)** Mice after CRS spent more time in the center area of the OFT. Unpaired two-tailed Student’s *t*-test, *t*_(28)_ = 2.899, *p* = 0.0072. **(G)** Control and CRS mice showed no difference in total distance. Unpaired two-tailed Student’s *t*-test, *t*_(28)_ = 0.5412, *p* = 0.5927. **(H)** Representative tracks of control and stress group in the elevated plus maze (EPM). **(I)** Mice after CRS spent less time in the open arms of EPM. Unpaired two-tailed Student’s *t*-test, *t*_(28)_ = 3.183, *p* = 0.0036. **(J)** Mice after CRS spent more time in the closed arms of EPM. Unpaired two-tailed Student’s *t*-test, *t*_(28)_ = 3.915, *p* < 0.001. **(K)** The mRNA expression of different mAChR subtypes in the vHPC in control and CRS mice. Unpaired two-tailed Student’s *t*-test. **(L)** The protein levels of M1 were increased in the vHPC of CRS mice. Unpaired two-tailed Student’s *t*-test, *t*_(6)_ = 5.097, *p* = 0.0022. The data are presented as the mean ± SEM, N.S.: not significant, *p < 0.05, **p < 0.01, ***p < 0.001. The numbers of mice are shown in parentheses.

We first assessed the impact of chronic stress on mouse behaviors. As described previously (Zhang et al., 2019), we used CRS to study the impact of stress on mice. Male mice were immobilized in the restrainer once for 2 h/day. After 10-day restraint, at the end of stress, emotional behaviors were assessed with the social interaction (SI), open-field test (OFT), and EPM test (Figure 1A); each behavior test was detected 1 day apart. We found that C57BL/6J mice showed social avoidance behavior after CRS, as they showed less desire for interaction in the SI test (Figures 1B,C; *F*_(1,56)_ = 5.631, *p* < 0.001) and showed a smaller SI ratio compared with no stress mice (Figure 1D; *t*_(28)_s = 4.086, *p* < 0.001). During a 5-min OFT, CRS mice spent less time in the center of the open field (Figures 1E,F; *t*_(28)_ = 2.899, *p* = 0.0072), although the total distance of CRS mice was not significantly different from that of the control group (Figure 1G; *t*_(28)_ = 0.5412, *p* = 0.5927). On the EPM, CRS mice in the CRS group showed less open-arm exploration (Figures 1H,I; *t*_(28)_ = 3.183, *p* = 0.0036) and more duration in closed arms (Figure 1J; *t*_(28)_ = 3.915, *p* < 0.001). These results suggest that CRS induces social avoidance and anxiety-like behaviors in C57BL/6J mice.

To investigate the change of mAChRs in vHPC, we detected mRNA and protein expression levels of mAChR subtypes in the vHPC. The qPCR experiments showed that CRS increased mRNA expression of M1 mAChRs and M3 mAChRs in the vHPC (Figure 1K; *t*_(12)_ = 6.019, *p* < 0.001, *t*_(12)_ = 2.951, *p* = 0.0121). Moreover, Western blotting experiments showed that CRS enhanced protein expression levels of M1 mAChRs (Figure 1L; ss*t*_(6)_ = 5.097, *p* = 0.0022).

### Transient Restraint Stress Increases Ventral Hippocampus Acetylcholine Muscarinic Receptor Signal in Behaving Mice

**Figure 2:**
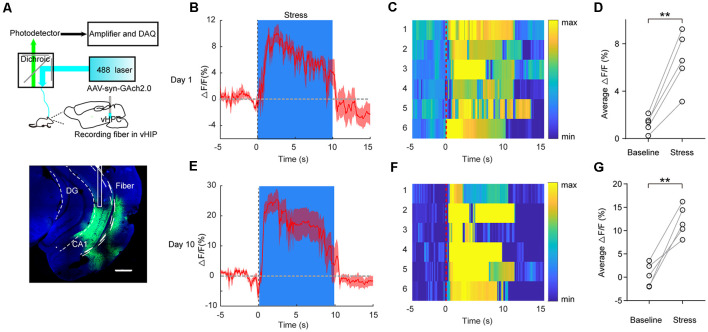
Transient restraint stress increases ACh muscarinic receptor (mAChR) signal in behaving mice. **(A)** Left, schematic of the fiber photometry setup. Right, a representative image validates GAch2.0 expression (green) and optical fiber tract above the vHPC. Scale bar, 500 μM. **(B,C)** PETH plot **(B)** and heat map **(C)** of fluorescence transients from a representative mouse aligned to the start of the restraint stress. **(D)** Quantification of change in fluorescence intensity before and after restraint stress (*N* = 5 mice). Paired Student’s *t*-test, *t*_(4)_ = 7.754, *p* = 0.0015. PETH plot **(E)** and heat map **(F)** of fluorescence transients from a representative mouse, which went through 9 days of CRS, aligned to the start of restraint stress on day 10. **(G)** Quantification of change in fluorescence intensity before and after restraint stress (*N* = 5 mice). Paired Student’s *t*-test, *t*_(4)_ = 6.399, *p* = 0.0031. The data are presented as the mean ± SEM, **p < 0.01. The numbers of mice are shown in parentheses.

A previous study had developed a fluorescent ACh indicator GAch2.0 based on mAChRs (Jing et al., 2018). Here, we injected AAV-syn-GAch2.0 virus into the vHPC and recorded GAch2.0 fluorescence signal using fiber photometry (Figure 2A). For the convenience of repeat recording within individuals, mice were gently held by the experimenter for 10 s using the right hand to imitate transient restraint stress. During the restraint test, we observed a rapid increase in fluorescence intensity when mice were restrained by hand (Figures 2B–D; *t*_(4)_ = 7.754, *p* = 0.0015). To test the effect of CRS on vHPC ACh release, the mice went through the CRS for 9 days, and on day 10, we recorded fluorescence intensity when mice were restrained by hand. Compared with the acute stress on day 1, after CRS, the stress induced more ACh release in the vHPC by showing a larger fluorescence intensity (Figures 2E–G; *t*_(4)_ = 6.399, *p* = 0.0031). This result indicated that restraint stress increased ACh release in the vHPC.

### Chronic Restraint Stress Increases Ventral Hippocampal Neuronal Activity by Enhancing Excitatory Synaptic Activity and Decreasing Inhibitory Synaptic Activity

**Figure 3:**
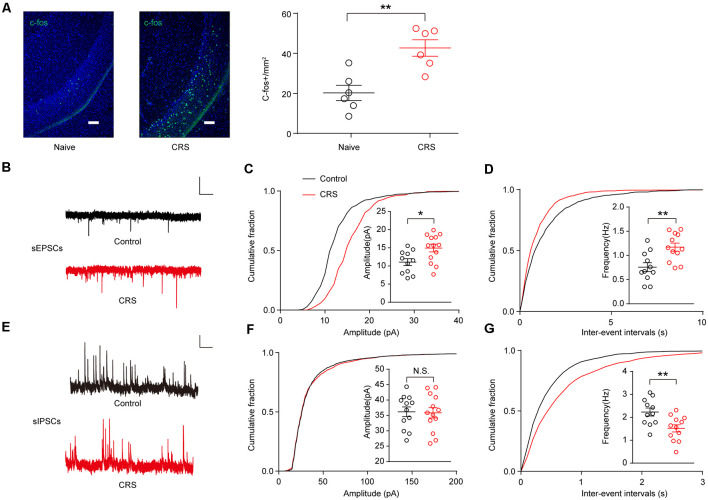
CRS increases ventral hippocampal activity by enhancing excitatory postsynaptic currents (EPSCs) and decreasing inhibitory postsynaptic currents (IPSCs). **(A)** Representative images of c-Fos (green) staining in the vHPC. Scale bar, 100 μM (Left). Quantification of vHPC c-Fos+ cells after CRS (Right). Unpaired two-tailed Student’s *t*-test, *t*_(10)_ = 4.013, *p* = 0.0025. **(B)** Representative spontaneous EPSC (sEPSC) traces of neurons in vHPC. Scale bar: 10 pA, 1 s. **(C)** Cumulative distribution of sEPSC amplitudes and average amplitudes. Unpaired two-tailed Student’s *t*-test, *t*_(22)_ = 2.685, *p* = 0.0135. **(D)** Cumulative distribution of sEPSC inter-event intervals and average frequencies. Unpaired two-tailed Student’s *t*-test, *t*_(21)_ = 3.337, *p* = 0.0031. **(E)** Representative spontaneous IPSC (sIPSC) traces of neurons in vHPC. Scale bar: 10 pA, 1 s. **(F)** Cumulative distribution of sIPSC amplitudes and average amplitudes. Unpaired two-tailed Student’s *t*-test, *t*_(23)_ = 0.1448, *p* = 0.8862. **(G)** Cumulative distribution of sIPSC inter-event intervals and average frequencies. Unpaired two-tailed Student’s *t*-test, *t*_(21)_ = 3.027, *p* = 0.0064. The data are presented as the mean ± SEM, N.S.: not significant, *p < 0.05, **p < 0.01. The numbers of recording neurons are shown in parentheses.

Next, we examine the effect of CRS on vHPC neuronal activity. C-Fos is widely used as a marker for neuronal activity. We first examined the c-Fos expression in the vHPC. Indeed, more than two-fold increase of c-Fos expression was found in the vHPC of CRS mice (Figure 3A; *t*_(10)_ = 4.013, *p* = 0.0025). To determine synaptic alterations triggered by CRS, we recorded sEPSCs and spontaneous inhibitory postsynaptic currents (sIPSCs) in vHPC pyramidal neurons by whole-cell patch-clamp recording of acute brain slices from control and CRS mice. We found that both the amplitude and frequency of sEPSCs significantly increased in CRS mice compared to those in control mice (Figures 3B,C; *t*_(22)_ = 2.685, *p* = 0.0135; Figure 3D; *t*_(21)_ = 3.337, *p* = 0.0031). Meanwhile, the frequency of sIPSCs but not the amplitude greatly decreased in CRS mice (Figures 3E,F; *t*_(23)_ss = 0.1448, *p* = 0.8862; Figure 3G; *t*_(21)_ = 3.027, *p* = 0.0064). Together, these c-Fos data and electrophysiological measurements showed that CRS increased neuronal activity and excitatory synaptic transmission but decreased inhibitory synaptic transmission in the vHPC pyramidal neurons.

### Scopolamine Reverses the Enhanced Spontaneous Excitatory Postsynaptic Currents in Ventral Hippocampus Neurons and Rescues Chronic Restraint Stress-Induced Mouse Social Avoidance and Anxiety-Like Behaviors

**Figure 4:**
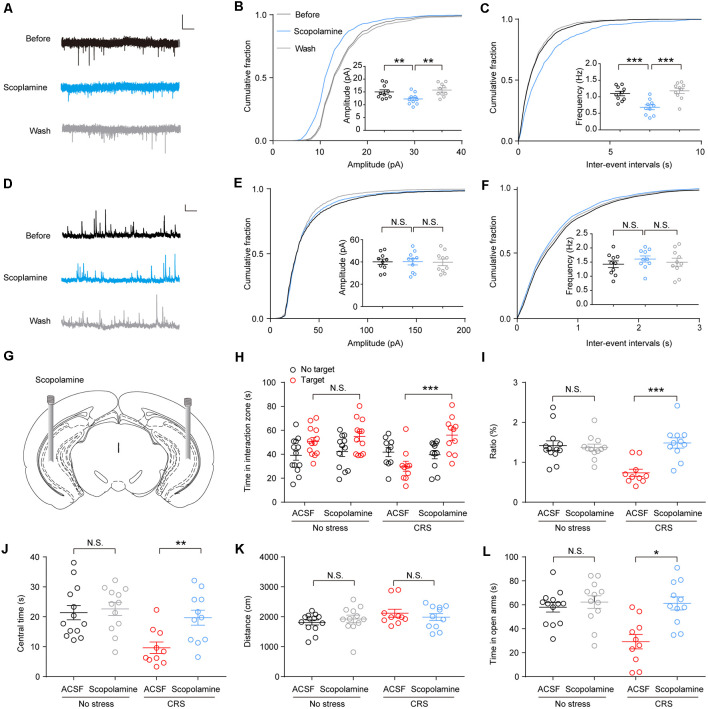
Scopolamine reverses the enhanced spontaneous excitatory postsynaptic currents (sEPSCs) in vHPC neurons and rescues CRS-induced mouse social avoidance and anxiety-like behaviors. **(A)** Representative sEPSC traces before, during, and after wash of scopolamine. Scale bar: 10 pA, 1 s. **(B)** Cumulative distribution of sEPSC amplitudes and average amplitudes. One-way ANOVA/least square difference (LSD) *post hoc* test. *F*_(1.619,14.57)_ = 18.99, *p* = 0.0002. Before vs. scopolamine, *t*_(9)_ = 4.369, *p* = 0.0036. Wash vs. scopolamine, *t*_(9)_ = 5.028, *p* = 0.0014. **(C)** Cumulative distribution of sEPSC inter-event intervals and average frequencies. One-way ANOVA/LSD *post hoc* test. *F*_(1.283,11.55)_ = 30.79, *p* < 0.0001. Before vs. scopolamine, *t*_(9)_ = 5.376, *p* = 0.0009. Wash vs. scopolamine, *t*_(9)_ = 6.081, *p* = 0.0004. **(D)** Representative spontaneous inhibitory postsynaptic current (sIPSC) traces before, during, and after wash of scopolamine. Scale bar: 10 pA, 1 s. **(E)** Cumulative distribution of sIPSC amplitudes and average amplitudes. One-way ANOVA/LSD *post hoc* test. *F*_(1.878,16.9)_ = 0.009277, *p* = 0.9882. Before vs. scopolamine, *t*_(9)_ = 0.0377, *p* > 0.9999. Wash vs. scopolamine, *t*_(9)_ = 0.1537, *p* > 0.9999. **(F)** Cumulative distribution of sIPSC inter-event intervals and average frequencies. One-way ANOVA/LSD *post hoc* test. *F*_(1.839,16.55)_ = 0.5085, *p* = 0.5954. Before vs. scopolamine, *t*_(9)_ = 0.9347, *p* = 0.7487. Wash vs. scopolamine, *t*_(9)_s = 0.5731, *p* > 0.9999. **(G)** Cartoon shows drug delivery in vHPC. **(H)** SI time in the absence or presence of social target. Two-way ANOVA/LSD *post hoc* test, *F*_(1,84)_ = 5.414, *p* = 0.022, target^ACSF + stress^ vs. target^scopolamine + stress^, *t* = 4.466, *p* < 0.001). **(I)** The ratio in the SI test. Two-way ANOVA/LSD *post hoc* test. *F*_(1,42)_ = 13.28, *p* < 0.001. CRS + artificial cerebrospinal fluid (ACSF) vs. CRS + scopolamine, *t* = 4.651, *p* < 0.0001. **(J)** The time spent in the center of OFT. Two-way ANOVA/LSD *post hoc* test. *F*_(1,42)_ = 3.671, *p* = 0.0621. CRS + ACSF vs. CRS + scopolamine, *t* = 2.975, *p* = 0.0097. **(K)** Mice total distance in OFT. Two-way ANOVA/LSD *post hoc* test. *F*_(1,42)_ = 1.414, *p* = 0.291. CRS + ACSF vs. CRS + scopolamine, *t* = 0.7562, *p* = 0.7016. **(L)** The time spent in the open arms of elevated plus maze (EPM). Two-way ANOVA/LSD *post hoc* test. *F*_(1,42)_ = 7.212, *p* = 0.0103. CRS + ACSF vs. CRS + scopolamine, *t* = 3.935, *p* = 0.015. The data are presented as the mean ± SEM, N.S.: not significant, *p < 0.05, **p < 0.01, ***p < 0.001. The numbers of recording neurons are shown in parentheses.

Scopolamine was a nonselective mAChR antagonist; previous studies demonstrate that it can produce rapid antidepressant actions (within hours) and is effective even in treatment-resistant major depressive disorder (MDD) patients (Furey et al., 2010; Drevets et al., 2013; Wohleb et al., 2016). We tested the effects of scopolamine (50 μM; Zhu et al., 2013) on synaptic transmission in the vHPC pyramidal neurons of CRS mice and found that bath perfusion of scopolamine significantly reduced the amplitude and frequency of sEPSCs (Figures 4A,B; *F*_(1.619,14.57)_ = 18.99, *p* = 0.0002; Figure 4C; *F*_(1.283,11.55)_ = 30.79, *p* < 0.0001). However, the delivery of scopolamine did not change the amplitude and frequency of sIPSCs of vHPC pyramidal neurons (Figures 4D,E; *F*_(1.878,16.9)_ = 0.009277, *p* = 0.9882; Figure 4F; *F*_(1.839,16.55)_ = 0.5085, *p* = 0.5954).

To confirm the effect of scopolamine on the behaviors in CRS mice, we infused scopolamine (50 μM, 0.5 μl/side) in the vHPC bilaterally (Figure 4G). Then, 30 min after scopolamine delivery, the CRS mice showed a greater desire for SI as they spent more time in the SI zone and a higher SI ratio (Figure 4H; *F*_(1,84)_ = 5.414, *p* = 0.22; Figure 4I; *F*_(1,42)_ = 13.28, *p* < 0.001). Furthermore, in the OFT, we found that intra-vHPC administration of scopolamine increased center time without affecting locomotion of CRS mice (Figure 4J; *F*_(1,42)_ = 3.671, *p* = 0.0621; Figure 4K; *F*_(1,42)_ = 1.141, *p* = 0.291). Moreover, the same treatment decreased open arms avoidance in CRS mice, as they spent more time in the open arms (Figure 4L; *F*_(1,42)_ = 7.212, *p* = 0.0103).

### M1 Knockdown in the Ventral Hippocampus Reverses Anxiogenic Effect of Chronic Restraint Stress

**Figure 5:**
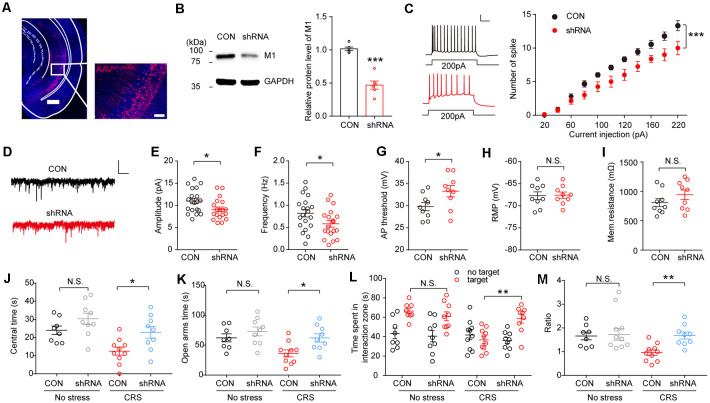
M1 knockdown in the vHPC reverses anxiogenic effect of CRS. **(A)** Representative confocal images of AAV-U6-M1shRNA-CMV-RFP (red) distribution in the vHPC. Scale bar, 500 μM; inset, 100 μM. **(B)** The protein levels of M1 were decreased in the vHPC of mice injected with an AAV expressing M1-shRNA. Unpaired two-tailed Student’s *t*-test, *t*_(10)_ = 7.269, *p* < 0.001. **(C)** Left, spikes elicited after 200 pA current injection in vHPC neurons. Scale bar, 20 pA, 100 ms. Right, spikes elicited in vHPC neurons in response to current injection. Two-way ANOVA/LSD *post hoc* test. *F*_(1,264)_ = 47.91, *p* < 0.001. **(D)** Representative spontaneous excitatory postsynaptic currents (sEPSCs) traces of neurons in vHPC. **(E,F)** M1 knockdown decreased sEPSCs amplitude **(E)**, Unpaired two-tailed Student’s *t*-test, *t*_(36)_ = 2.241, *p* = 0.0313, and frequency of vHPC neurons. Unpaired two-tailed Student’s *t*-test, *t*_(36)_ = 2.039, *p* = 0.0489. **(G–I)** a.p. threshold, resting membrane potential (RMP) and membrane resistance for vHPC neurons. Unpaired two-tailed Student’s *t*-test, *t*_(16)_ = 2.16, *p* = 0.0463. Scale bar, 10 pA, 1 s. **(J)** The time spent in the center of the OFT. Two-way ANOVA/LSD *post hoc* test. *F*_(1,32)_ = 0.5203, *p* = 0.4760. CRS + CON vs. CRS + shRNA, *t* = 2.705, *p* = 0.0217. **(K)** The time spent in the open arms of the elevated plus maze (EPM). Two-way ANOVA/LSD *post hoc* test. *F*_(1,32)_ = 1.243, *p* = 0.2732. CRS + CON vs. CRS + shRNA, *t* = 2.784, *p* = 0.0179. **(L)** SI time in the absence or presence of a social target. Two-way ANOVA/LSD *post hoc* test, *F*_(1,64)_ = 4.5, *p* = 0.0063, target^CON + stress^ vs. target^CON + stress^, *t* = 3.547, *p* = 0.0044. **(M)** The ratio in the SI test. Two-way ANOVA/LSD *post hoc* test. *F*_(1,32)_ = 5.142, *p* = 0.0302. CRS + CON vs. CRS + shRNA, *t* = 3.04, *p* = 0.0094. The data are presented as the mean ± SEM, N.S.: not significant, *p < 0.05, **p < 0.01, ***p < 0.001. The numbers of mice are shown in parentheses.

To further confirm the role of the vHPC M1 mAChRs in the genesis of anxiety behaviors in mice, M1 mAChRs in the vHPC were knocked down using the adeno-associated virus expressing M1-shRNA injected into the vHPC (Figure 5A). Using immunoblotting, we confirmed a high efficiency of M1 knockdown in the vHPC of mice after AAV-U6-M1shRNA-CMV-RFP injection (Figure 5B; *t*_(10)_ = 7.269, *p* < 0.001). *Ex vivo* acute brain slices were prepared, and whole-cell patch-clamp recordings were obtained from mice injected with the virus in vHPC. Interestingly, we observed a decrease in intrinsic excitability of vHPC neurons after interference of M1 expression (Figure 5C; *F*_(1,264)_ = 47.91, *p* < 0.001). Meanwhile, we examined changes in synaptic inputs by measuring sEPSCs and found M1 knockdown in the vHPC of mice had significantly smaller amplitude and frequency of excited synaptic inputs (Figures 5D,E; *t*_(36)_ = 2.241, *p* = 0.0313; Figure 5F; *t*_(36)_ = 2.039, *p* = 0.0489). Changes in synaptic input may cause excitability changes. In order to isolate excitability indicators from synaptic inputs, we measured intrinsic excitability in the presence of excitability and inhibitory synaptic current blockers NBQX and methiodinucleotide (BMI). We found that the threshold is significantly increased (Figure 5G; *t*_(16)_ = 2.16, *p* = 0.0463), while the resting membrane potential (RMP) and membrane resistance remained unchanged (Figures 5H,I); this suggests that the intrinsic excitability changes are not secondary effects of synaptic changes. Then, AAV-U6-M1shRNA-CMV-RFP virus and the control virus were injected into two groups of C57 mice. The mice were performed CRS 2 weeks after viral injection before behavioral tests. We observed that mice with M1 knockdown in the vHPC spent more time in the center in the OFT (Figure 5J; *F*_(1,32)_ = 0.5230, *p* = 0.4760) and time spent in the open arms in the EPM test (Figure 5K; *F*_(1,32)_ = 1.243, *p* = 0.2732) in comparison with the control group. In the SI test, vHPC M1 knockdown mice show more desire for the interaction with a strange mouse (Figure 5L; *F*_(1,64)_ = 4.5, *p* = 0.0063; Figure 5M; *F*_(1,32)_ = 5.142, *p* = 0.0302). These results show that M1 mAChR expression in the vHPC is critically required for the development of stress-induced anxiety, consistent with those observed in mice infused with scopolamine in the vHPC.

## Discussion

In the present study, the major findings are as follows. Chronic stress induced social avoidance and anxiety-like behavior and increased mAChR expression in C57BL/6J mice. Transient restraint stress increased ACh release in the vHPC. Electrophysiological measurements indicated that CRS enhanced the amplitude and frequency of sEPSCs while decreasing the frequency of sIPSCs in ventral hippocampal neurons. The mAChR antagonist scopolamine attenuated the excitatory synaptic activity in vHPC neuron and rescued the social avoidance and anxiety-like behavior induced by CRS in mice. Furthermore, M1 receptor knockdown in the vHPC reversed the anxiogenic effect of CRS in mice. To our knowledge, this is the first study to demonstrate that ventral hippocampal mAChRs are critical for stress-induced anxiety-like behavior in mice.

Hippocampus, especially the vHPC, is intimately tied to emotion. The vHPC modulates stress responses, and its dysfunction leads to affective disorders such as anxiety (Fanselow and Dong, 2010; Parfitt et al., 2017; Jimenez et al., 2018). Previous studies found that ventral hippocampal neurons control anxiety by their projection to medial prefrontal cortex (mPFC), lateral septum, and lateral hypothalamic area. The vHPC modulates anxiety-like behavior through influencing neuronal activities (Tu et al., 2014; Fisher et al., 2017; Rytova et al., 2019). In our experiments, chronic stress induced anxiety-like behaviors and enhanced neuronal activities in vHPC in mice. This is consistent with several previous findings that activation of vHPC neurons elicits negative emotions, including anxiety in mice (Bannerman et al., 2003; Felix-Ortiz et al., 2013; Padilla-Coreano et al., 2016; Jimenez et al., 2018).

The hippocampus, both the dorsal and ventral regions, receives cholinergic fiber from basal forebrain cholinergic neurons, especially the medial septum (Li et al., 2018). ACh released from these neurons alters neuronal excitability, influences synaptic transmission, induces synaptic plasticity, and coordinates firing of groups of neurons (Picciotto et al., 2012). Throughout the hippocampus, M1 receptor is mainly expressed in the pyramidal cell bodies and dendrites. The most consistent effect of exogenously applied ACh to hippocampal pyramidal neurons is membrane depolarization with increased membrane resistance based on the electrophysiological experiment (Cole and Nicoll, 1984). However, in our study, interference of M1 receptor did not influence membrane resistance (Figures 5H,I), so, the impact of ACh on membrane resistance may be through its activation of nicotine receptors or other mAChRs. Also, muscarinic activation excited pyramidal cells by regulating ion channels on neurons including the K^+^ currents I_M_ and I_AHP_ and the Ca^2+^-activated K^+^ currents responsible for slowing action potential discharges (Dutar et al., 1995). This may be the reason for AP threshold change after M1 knockdown in vHPC. Besides these, our study found that mAChRs can also influence ventral hippocampal neurons’ activities *via* synaptic level. This is consistent with the previous finding that in the dorsal CA1 region of the hippocampus, ACh released from cholinergic fibers modulates hippocampal synaptic plasticity through the postsynaptic M1 mAChR activation (Shinoe et al., 2005). Our *in vivo* data show that transient restraint stress induces ACh release in vHPC (Figure 2); this is consistent with previous studies showing that stress increases ACh level in the hippocampus (Mark et al., 1996; Dong et al., 2004). Furthermore, pharmacological and molecular genetic decreases in hippocampal AChE activity increased anxiety-like behaviors (Mineur et al., 2013; Fernandes et al., 2018). Our study indicated that the ACh modulates mouse emotion behavior *via* mAChRs.

Furthermore, we found that scopolamine rescued stress-induced abnormal behavior by decreasing sEPSCs but not affecting sIPSCs in vHPC neurons. These results indicate that scopolamine produces the inhibitory effects on anxiety behavior through M1 mAChRs on vHPC neurons. Our electrophysiological experiments showed that both the amplitude and frequency of sEPSCs were significantly increased in CRS mice, indicating an increased glutamate release from presynaptic terminals or enhanced response of postsynaptic glutamate receptors. We also found that the frequency of sIPSCs, but not the amplitude, was greatly decreased in CRS mice, indicating a reduced gamma aminobutyric acid (GABA) release from presynaptic terminals without altering the postsynaptic GABA receptors. Together, these data suggest that CRS produces stronger synaptic plasticity at the excitatory glutamatergic synapses than the inhibitory GABAergic synapses in vHPC neurons. Thus, the decrease of sEPSCs by scopolamine may be enough for mice to disengage from the stress. Further work needs to investigate how M1 mAChRs regulate sEPSCs.

Many psychiatric illnesses are characterized by deficits in the social domain (Allsop et al., 2014). A previous study found that amygdala–vHPC glutamatergic projection could bidirectionally modulate mouse SI (Felix-Ortiz and Tye, 2014). Moreover, relaxin-3 receptor on GABA neurons in rat vHPC modulates mouse social avoidance (Rytova et al., 2019). Interestingly, our study found that intra-vHPC injection of scopolamine and knockdown of M1 mAChRs in the vHPC can attenuate CRS mouse social avoidance. As the amygdala neurons’ activities show great change after chronic stress (Zhang et al., 2019), M1 mAChRs might regulate social behavior through the modulation of amygdala–vHPC glutamatergic transmission.

A series of studies suggest that scopolamine has antidepressant effects in depressed humans (Dulawa and Janowsky, 2019), shows promise for the treatment of opioid use disorder (Jensen et al., 2018), and has significant postoperative nausea and vomiting (PONV) therapeutic efficacy (Kassel et al., 2018). In this study, we found that scopolamine had a significant effect on social behavior in mice with chronic stress (Figures 4H,I). Notably, besides the above, scopolamine may benefit surgical patients because of its drying effects, resulting in fewer secretions and complications with intubation (Kassel et al., 2018). These suggest that scopolamine has potential for treating surgical patients with preoperative anxiety at age 18–65, considering some significant side effects in old patients and children.

In conclusion, this study reveals the functional role of the vHPC M1 mAChRs in stress-induced social avoidance and anxiety, which may provide new insight for treatments of anxiety disorders.

## Data Availability Statement

The original contributions presented in the study are included in the article, further inquiries can be directed to the corresponding author.

## Ethics Statement

The animal study was reviewed and approved by Institutional Laboratory Animal Care and Use Committee of Guangdong Provincial People’s Hospital.

## Author Contributions

HS designed the research. HS and LM analyzed the data and wrote the article. HL performed electrophysiological experiment. LM and YZ conducted the behavioral tests, and performed the virus injection. YS performed the western blots, qPCR and immunofluorescence. DZ performed fiber photometry. PL made contribution to the work of data collection and data analysis. All authors contributed to the article and approved the submitted version.

## Conflict of Interest

The authors declare that the research was conducted in the absence of any commercial or financial relationships that could be construed as a potential conflict of interest.

## References

[B1] Allsop S. A. Vander Weele C. M. Wichmann R. Tye K. M. (2014). Optogenetic insights on the relationship between anxiety-related behaviors and social deficits. Front. Behav. Neurosci. 8:241. 10.3389/fnbeh.2014.00241 25076878 PMC4099964

[B2] Bannerman D. M. Grubb M. Deacon R. M. Yee B. K. Feldon J. Rawlins J. N. (2003). Ventral hippocampal lesions affect anxiety but not spatial learning. Behav. Brain Res. 139, 197–213. 10.1016/s0166-4328(02)00268-1 12642189

[B3] Bannerman D. M. Yee B. K. Good M. A. Heupel M. J. Iversen S. D. Rawlins J. N. P. (1999). Double dissociation of function within the hippocampus: a comparison of dorsal, ventral, and complete hippocampal cytotoxic lesions. Behav. Neurosci. 113, 1170–1188. 10.1037/0735-7044.113.6.1170 10636297

[B4] Bartko S. J. Winters B. D. Saksida L. M. Bussey T. J. (2014). Different roles for M1 and M2 receptors within perirhinal cortex in object recognition and discrimination. Neurobiol. Learn. Mem. 110, 16–26. 10.1016/j.nlm.2014.01.002 24462721

[B5] Bluett E. J. Homan K. J. Morrison K. L. Levin M. E. Twohig M. P. (2014). Acceptance and commitment therapy for anxiety and OCD spectrum disorders: an empirical review. J. Anxiety Disord. 28, 612–624. 10.1016/j.janxdis.2014.06.008 25041735

[B6] Cole A. E. Nicoll R. A. (1984). Characterization of a slow cholinergic post-synaptic potential recorded *in vitro* from rat hippocampal pyramidal cells. J. Physiol. 352, 173–188. 10.1113/jphysiol.1984.sp015285 6747887 PMC1193205

[B7] Dasgupta R. Seibt F. Beierlein M. (2018). Synaptic release of acetylcholine rapidly suppresses cortical activity by recruiting muscarinic receptors in layer 4. J. Neurosci. 38, 5338–5350. 10.1523/JNEUROSCI.0566-18.2018 29739869 PMC5990982

[B8] Dong Y. Mao J. Shangguan D. Zhao R. Liu G. (2004). Acetylcholine release in the hippocampus during the operant conditioned reflex and the footshock stimulus in rats. Neurosci. Lett. 369, 121–125. 10.1016/j.neulet.2004.07.048 15450680

[B9] Drevets W. C. Zarate C. A. Jr. Furey M. L. (2013). Antidepressant effects of the muscarinic cholinergic receptor antagonist scopolamine: a review. Biol. Psychiatry 73, 1156–1163. 10.1016/j.biopsych.2012.09.031 23200525 PMC4131859

[B10] Dulawa S. C. Janowsky D. S. (2019). Cholinergic regulation of mood: from basic and clinical studies to emerging therapeutics. Mol. Psychiatry 24, 694–709. 10.1038/s41380-018-0219-x 30120418 PMC7192315

[B11] Dutar P. Bassant M. H. Senut M. C. Lamour Y. (1995). The septohippocampal pathway: structure and function of a central cholinergic system. Physiol. Rev. 75, 393–427. 10.1152/physrev.1995.75.2.393 7724668

[B12] Dias-Ferreira E. Sousa J. C. Melo I. Morgado P. Mesquita A. R. Cerqueira J. J.. (2009). Chronic stress causes frontostriatal reorganization and affects decision-making. Science 325, 621–625. 10.1126/science.1171203 19644122

[B13] Fanselow M. S. Dong H.-W. (2010). Are the dorsal and ventral hippocampus functionally distinct structures? Neuron 65, 7–19. 10.1016/j.neuron.2009.11.031 20152109 PMC2822727

[B14] Felder C. C. Goldsmith P. J. Jackson K. Sanger H. E. Evans D. A. Mogg A. J.. (2018). Current status of muscarinic M1 and M4 receptors as drug targets for neurodegenerative diseases. Neuropharmacology 136, 449–458. 10.1016/j.neuropharm.2018.01.028 29374561

[B16] Felix-Ortiz A. C. Beyeler A. Seo C. Leppla C. A. Wildes C. P. Tye K. M. (2013). BLA to vHPC inputs modulate anxiety-related behaviors. Neuron 79, 658–664. 10.1016/j.neuron.2013.06.016 23972595 PMC4205569

[B15] Felix-Ortiz A. C. Tye K. M. (2014). Amygdala inputs to the ventral hippocampus bidirectionally modulate social behavior. J. Neurosci. 34, 586–595. 10.1523/JNEUROSCI.4257-13.2014 24403157 PMC3870937

[B17] Fernandes S. S. Koth A. P. Parfitt G. M. Cordeiro M. F. Peixoto C. S. Soubhia A.. (2018). Enhanced cholinergic-tone during the stress induce a depressive-like state in mice. Behav. Brain Res. 347, 17–25. 10.1016/j.bbr.2018.02.044 29501509

[B18] Fisher M. L. Lemalefant R. M. Zhou L. Huang G. Turner J. R. (2017). Distinct roles of CREB within the ventral and dorsal hippocampus in mediating nicotine withdrawal phenotypes. Neuropsychopharmacology 42, 1599–1609. 10.1038/npp.2016.257 27848935 PMC5518892

[B19] Furey M. L. Khanna A. Hoffman E. M. Drevets W. C. (2010). Scopolamine produces larger antidepressant and antianxiety effects in women than in men. Neuropsychopharmacology 35, 2479–2488. 10.1038/npp.2010.131 20736989 PMC3055321

[B20] Hasselmo M. E. (2006). The role of acetylcholine in learning and memory. Curr. Opin. Neurobiol. 16, 710–715. 10.1016/j.conb.2006.09.002 17011181 PMC2659740

[B21] Jensen K. P. Devito E. E. Yip S. Carroll K. M. Sofuoglu M. (2018). The cholinergic system as a treatment target for opioid use disorder. CNS Drugs 32, 981–996. 10.1007/s40263-018-0572-y 30259415 PMC6314885

[B22] Jimenez J. C. Su K. Goldberg A. R. Luna V. M. Biane J. S. Ordek G.. (2018). Anxiety cells in a hippocampal-hypothalamic circuit. Neuron 97, 670.e6–683.e6. 10.1016/j.neuron.2018.01.016 29397273 PMC5877404

[B23] Jing M. Zhang P. Wang G. Feng J. Mesik L. Zeng J.. (2018). A genetically encoded fluorescent acetylcholine indicator for *in vitro* and *in vivo* studies. Nat. Biotechnol. 36, 726–737. 10.1038/nbt.4184 29985477 PMC6093211

[B24] Kassel L. Nelson M. Shine J. Jones L. R. Kassel C. (2018). Scopolamine use in the perioperative patient: a systematic review. AORN J. 108, 287–295. 10.1002/aorn.12336 30156728

[B25] Li X. Yu B. Sun Q. Zhang Y. Ren M. Zhang X.. (2018). Generation of a whole-brain atlas for the cholinergic system and mesoscopic projectome analysis of basal forebrain cholinergic neurons. Proc. Natl. Acad. Sci. U S A 115, 415–420. 10.1073/pnas.1703601115 29259118 PMC5777024

[B26] Mark G. P. Rada P. Shors T. J. (1996). Inescapable stress enhances extracellular acetylcholine in the rat hippocampus and prefrontal cortex but not the nucleus accumbens or amygdala. Neuroscience 74, 767–774. 10.1002/acn3.51249 8884772

[B28] McEwen B. S. Bowles N. P. Gray J. D. Hill M. N. Hunter R. G. Karatsoreos I. N.. (2015). Mechanisms of stress in the brain. Nat. Neurosci. 18, 1353–1363. 10.1038/nn.4086 26404710 PMC4933289

[B27] McEwen B. S. Morrison J. H. (2013). The brain on stress: vulnerability and plasticity of the prefrontal cortex over the life course. Neuron 79, 16–29. 10.1016/j.neuron.2013.06.028 23849196 PMC3753223

[B29] Mineur Y. S. Obayemi A. Wigestrand M. B. Fote G. M. Calarco C. A. Li A. M.. (2013). Cholinergic signaling in the hippocampus regulates social stress resilience and anxiety- and depression-like behavior. Proc. Natl. Acad. Sci. U S A 110, 3573–3578. 10.1073/pnas.1219731110 23401542 PMC3587265

[B30] Padilla-Coreano N. Bolkan S. S. Pierce G. M. Blackman D. R. Hardin W. D. Garcia-Garcia A. L.. (2016). Direct ventral hippocampal-prefrontal input is required for anxiety-related neural activity and behavior. Neuron 89, 857–866. 10.1016/j.neuron.2016.01.011 26853301 PMC4760847

[B31] Parfitt G. M. Nguyen R. Bang J. Y. Aqrabawi A. J. Tran M. M. Seo D. K.. (2017). Bidirectional control of anxiety-related behaviors in mice: role of inputs arising from the ventral hippocampus to the lateral septum and medial prefrontal cortex. Neuropsychopharmacology 42, 1715–1728. 10.1038/npp.2017.56 28294135 PMC5518909

[B32] Picciotto M. R. Higley M. J. Mineur Y. S. (2012). Acetylcholine as a neuromodulator: cholinergic signaling shapes nervous system function and behavior. Neuron 76, 116–129. 10.1016/j.neuron.2012.08.036 23040810 PMC3466476

[B33] Rytova V. Ganella D. E. Hawkes D. Bathgate R. A. D. Ma S. Gundlach A. L. (2019). Chronic activation of the relaxin-3 receptor on GABA neurons in rat ventral hippocampus promotes anxiety and social avoidance. Hippocampus 29, 905–920. 10.1002/hipo.23089 30891856

[B34] Sandi C. Haller J. (2015). Stress and the social brain: behavioural effects and neurobiological mechanisms. Nat. Rev. Neurosci. 16, 290–304. 10.1038/nrn3918 25891510

[B35] Shinoe T. Matsui M. Taketo M. M. Manabe T. (2005). Modulation of synaptic plasticity by physiological activation of M1 muscarinic acetylcholine receptors in the mouse hippocampus. J. Neurosci. 25, 11194–11200. 10.1523/JNEUROSCI.2338-05.2005 16319319 PMC6725656

[B36] Tao Y. Chen Y. J. Shen C. Luo Z. Bates C. R. Lee D.. (2013). Erbin interacts with TARP gamma-2 for surface expression of AMPA receptors in cortical interneurons. Nat. Neurosci. 16, 290–299. 10.1038/nn.3320 23354328

[B37] Thomsen M. Wörtwein G. Fink-Jensen A. Woldbye D. P. D. Wess J. Caine S. B. (2007). Decreased prepulse inhibition and increased sensitivity to muscarinic, but not dopaminergic drugs in M5 muscarinic acetylcholine receptor knockout mice. Psychopharmacology 192, 97–110. 10.1007/s00213-006-0682-y 17310388

[B38] Tu W. Cook A. Scholl J. L. Mears M. Watt M. J. Renner K. J.. (2014). Serotonin in the ventral hippocampus modulates anxiety-like behavior during amphetamine withdrawal. Neuroscience 281, 35–43. 10.1016/j.neuroscience.2014.09.019 25241066 PMC4364927

[B39] Volpicelli L. A. Levey A. I. (2004). Muscarinic acetylcholine receptor subtypes in cerebral cortex and hippocampus. Prog. Brain Res. 145, 59–66. 10.1016/S0079-6123(03)45003-6 14650906

[B40] Weeden C. S. Roberts J. M. Kamm A. M. Kesner R. P. (2015). The role of the ventral dentate gyrus in anxiety-based behaviors. Neurobiol. Learn. Mem. 118, 143–149. 10.1016/j.nlm.2014.12.002 25498221

[B41] Wess J. Eglen R. M. Gautam D. (2007). Muscarinic acetylcholine receptors: mutant mice provide new insights for drug development. Nat. Rev. Drug Discov. 6, 721–733. 10.1038/nrd2379 17762886

[B42] Wohleb E. S. Wu M. Gerhard D. M. Taylor S. R. Picciotto M. R. Alreja M.. (2016). GABA interneurons mediate the rapid antidepressant-like effects of scopolamine. J. Clin. Invest. 126, 2482–2494. 10.1172/JCI85033 27270172 PMC4922686

[B43] Zhang J. Y. Liu T. H. He Y. Pan H. Q. Zhang W. H. Yin X. P.. (2019). Chronic stress remodels synapses in an amygdala circuit-specific manner. Biol. Psychiatry 85, 189–201. 10.1016/j.biopsych.2018.06.019 30060908 PMC6747699

[B44] Zhu Y. Y. Jing L. Duan T. T. Yuan Q. Cao J. Zhou Q. X.. (2013). Patterned high-frequency stimulation induces a form of long-term depression dependent on GABA_A_ and mACh receptors in the hippocampus. Neuroscience 250, 658–663. 10.1016/j.neuroscience.2013.07.059 23911810

